# Impact of high proviral load on milk production, reproduction and subclinical diseases in dairy cows infected with bovine leukemia virus

**DOI:** 10.3389/fvets.2025.1522089

**Published:** 2025-03-05

**Authors:** Simon Bourassi, Shawn McKenna, Greg Keefe, Emily John, John VanLeeuwen, Emilia Bourassi, J. Trenton McClure

**Affiliations:** ^1^Department of Health Management, Atlantic Veterinary College, University of Prince Edward Island, Charlottetown, PE, Canada; ^2^Department of Companion Animals, Atlantic Veterinary College, University of Prince Edward Island, Charlottetown, PE, Canada

**Keywords:** BLV, proviral load, dairy cows, bovine leukosis, milk production, mastitis, reproduction

## Abstract

**Introduction:**

Bovine Leukemia Virus (BLV) prevalence remains high in dairy cattle in North America. Quantifying the proviral load (PVL) in BLV-positive cows can be used to control this disease in herds where BLV is prevalent by focusing culling of high PVL animals to reduce the risk of transmission. The impact of high BLV PVL on dairy cows’ performance is not well established. The objective of this study was to assess the effect of high PVL status on milk production, occurrence of subclinical ketosis or mastitis, or fertility in BLV-infected cows.

**Methods:**

Twenty-five herds from the three Maritime provinces in Atlantic Canada were enrolled in this study. BLV infected cows were first identified by individual milk or serum testing. A validated quantitative qPCR was used to quantify the PVL in cows with positive BLV antibody results. Parity, 305-day milk production, annual geometric average somatic cell count, fat-to-protein ratio in milk on the first test post-calving, days in milk at first service, and calving-to-conception interval were collected from DairyComp305 software. Two-level mixed multivariable regression models were used to assess the relationship between BLV PVL and milk production, subclinical mastitis and ketosis and reproduction performance.

**Results:**

High PVL was strongly associated with reduced milk production (387 kg and 431 kg) and reproduction performance (calving-to-conception interval lengthened by 50 days and 49 days), and higher odds of subclinical mastitis (Odds ratio = 2.38 and 2.48), when compared to BLVpositive cows with a low PVL and BLV-negative cows, respectively.

**Conclusion:**

These results support implementing a control program to prioritize culling high PVL cows.

## Introduction

Bovine Leukemia Virus (BLV) is a lymphotropic deltaretrovirus responsible for Enzootic Bovine Leukosis (1, 2). It integrates primarily into B lymphocytes, where it creates a provirus, causing a lifelong persistent infection (3). Most BLV-infected cows appear clinically asymptomatic while 30% develop a persistent lymphocytosis and less than 5% develop B-cell lymphoma (4, 5).

Although eradicated in most countries in Europe, BLV prevalence in dairy cattle in North America and other parts of the world remains high, with prevalences in North America averaging 90 and 40% for herd-level and within-herd prevalences, respectively (6). Given that there is no treatment for this disease, and there is no commercially available and validated vaccine, preventing transmission is paramount to controlling the spread of the virus (7, 8). The high prevalence warrants new management strategies to control this disease, as detecting and culling all BLV-infected cattle is not economically feasible (9). Recent research has focused on proviral load (PVL). Proviral load is the number of copies of viral genome integrated into the host genome in B-lymphocytes and is measured using quantitative PCR (10, 11). It appears that cows with low PVL are unlikely to be a source of infection for BLV-negative cows, which means that culling only cows with high PVL could be a reasonable control strategy in herds with high BLV prevalence (9, 12). This strategy is corroborated by a recent study showing that transmission rates in BLV-infected cows with persistent lymphocytosis, which has been shown to correlate with high proviral loads, are approximately 70 times higher than in aleukemic BLV infected cows (13).

There is controversy concerning the impact that BLV has in the dairy industry, with some studies demonstrating negative effects of BLV on production, longevity and other parameters, while others could not find a significant association (14–16). It has been found that BLV infection can lead to significant economic losses through reduction in milk production, fertility, and lifespan, impairment of the immune system, as well as negative impacts on international trade and carcass condemnation (17–20). The overall annual economic loss in the dairy industry in the United States was estimated to be around $285 million for producers and $525 million for the entire industry (21). A recent study in Canada showed an estimated loss of herd-based partial net revenue of $92, 587 per year in a herd of 146 animals (18).

Although reasons for these inconsistent findings on productivity and economic impact are probably multifactorial, differences in the prevalence of cows with a high proviral load could contribute to these discrepancies. There are only a few studies looking at the role of BLV PVL in production losses, with a recent study showing a negative impact on milk production (22).

No study has looked at both milk production and reproduction parameters, as well as subclinical diseases on a same population. If negative associations between cows with a high BLV PVL are found with milk and other production measures compared to cows that are BLV positive but with a low PVL, this could be an additional incentive for producers to measure PVL to determine which BLV-positive cows to cull, in herds with high prevalence.

The objective of this study was to evaluate if there is an association between BLV PVL status (high vs. low) and milk production, fertility, and occurrence of subclinical mastitis and subclinical ketosis.

## Materials and methods

### Study design

This was a cross-sectional study.

### Herd selection

Dairy producers from the three Canadian Maritime provinces (New Brunswick, Nova Scotia and Prince Edward Island) volunteered to enroll their herds in this study. Due to research budget constraints, enrollment was limited to 30 herds. Inclusion criteria included the following: (1) willingness to participate during the entire study period; (2) herd registered in Dairy Herd Improvement (DHI) monthly milk quality monitoring; and (3) implementation of good management practices to prevent transmission of BLV, including use of one single-use needle per animal, one rectal sleeve per animal and a farm method of fly control. Enrolled herds consented for the Maritime Quality Milk (MQM) laboratory to use milk collected for routine milk testing by DHI technicians, as well as collection of blood samples via venipuncture by their herd veterinarian. They also consented to provide researchers access to their herd and individual cows records through DairyComp305 software (DC305) (Valley Agricultural Software, Tulare, California, USA) during the study period. This study was approved by the Animal Care and Use Committee of the University of Prince Edward Island (File #6008434).

### Sampling and laboratory analysis

All sampling and testing were performed between February and March 2021 in two stages.

#### Stage 1: Anti-BLV antibody ELISA testing

Approximately 30 mL of milk were collected in standard milk sample cups from each lactating cow and transferred from the DHI laboratory to the MQM laboratory. Samples were collected by the DHI technician, preserved with one BROTAB milk preservative tablet (Sierra Court, CA, USA), and transported in a cooler at 4°C. These samples were also kept at 4°C at the DHI milk testing laboratory until transfer to the MQM laboratory where they were also refrigerated until ELISA testing for BLV antibodies was performed, typically within one week from the time of collection.

Blood samples from dry cows and pregnant heifers were collected by herd veterinarians, transported on ice, and separated into serum by spinning at 3000 *g* for 15 min. The serum was frozen and stored at −20°C until analysis at the MQM laboratory.

An antibody ELISA test (Bovicheck BLV ELISA kit TRM-506, Biovet inc., St Hyacinthe, QC, Canada) for anti-gp51 antibodies to BLV was performed on serum and milk samples following the manufacturer’s instructions. Individuals were classified as BLV-positive (BLV^+^), BLV-negative (BLV^−^) or BLV-suspect according to the manufacturer’s cutoffs (inhibition percentage > 30% and > 45% for milk and serum, respectively, for BLV^+^, < 20% and < 35% for milk and serum, respectively, for BLV^−^; with values in between considered BLV-suspect). All BLV-suspects were retested with the ELISA test on serum 4 weeks after the first testing. All BLV-suspect animals were BLV-positive when retested.

#### Stage 2: qPCR BLV PVL quantification

Whole blood was collected in an EDTA vacutainer from all BLV^+^ cattle by the herd veterinarian and shipped to the MQM laboratory. DNA was extracted using the Qiagen DNEasy blood and tissue kit (Qiagen Inc. Montreal, Quebec, Canada), as described in a previous study (23). Briefly, 219 μL of buffer AL (lysis buffer) and 40 μL of proteinase K were added to 0.2 mL of serum in a 1.5 mL Eppendorf tube and pulse vortexed 10 times. After incubation of the tubes at 56°C for 15 min, 219 μL of pure ethanol was added to each tube and pulse vortexed 10 times before being centrifuged at 8000 g for 5 min. The collection tubes were replaced and 0.5 mL of solution AW1 (washer buffer) was added to each tube and centrifuged again at 8000 g for 5 min. The same process was repeated with 0.5 mL of solution AW2 (washer buffer) with a centrifugation at 16300 g for 10 min. The spin columns were then moved to new 1.5 mL Eppendorf tubes. Forty μL of solution AE (elution buffer) was added to each membrane and centrifuged at 8000 g for one minute. Extracted DNA concentration was determined using the NanoDrop™ 2000 Spectrophotometer (ThermoFisher Scientific™, Mississauga, Ontario) to ensure that the concentration was consistently >30 ng/μL. The samples were kept at −80°C until qPCR analysis.

BLV proviral load (PVL) was quantified for the extracted DNA samples using the BLV SS1 qPCR assay (CentralStar Cooperative Inc., East Lansing, MI), as described previously (23, 24). This assay is a multiplex probe-based quantitative PCR targeting the BLV polymerase and the bovine ß-Actin genes and containing a spike-in control to allow quantification of PVL. PCR components were 3 μL of DNA sample, 7.25 μL nuclease-free water, 12.5 μL 2XPrimeTime gene Expression Master Mix, 1.25 μL of 20X Primer Master Mix (BLV SS1 primer), and 1 μL of spike-in positive amplification control. All qPCR was performed on CFX96 BioRad touch Real-time PCR System (BioRad, Mississauga, Ontario) under the following conditions: 95°C for 3 min, 40 cycles of 95°C for 15 s and 60°C for 1 min, before a final 1 min at 60°C. Copy numbers of BLV and ß-Actin were calculated using standard curves. Proviral load was estimated by first dividing the copies of Bos actin by 2 (each cell with a nucleus contains 2 copies of the gene) to estimate the number of white blood cell (WBC) genomes amplified and then the number of BLV copies was divided by the estimated number of WBCs.

### Data collection

#### Herd level variables

Herd-level variables were retrieved from a questionnaire given to producers and included housing type (free-stall, tie-stall, other), herd size, herd predominant breed(s) and type of milking system (milk line, parlor, or robot).

#### Cow level variables

The cow-level variables retrieved from DC305 were as follow: (1) Parity at the time of testing, modeled as a categorical variable of 4 levels (1, 2, 3 and ≥ 4); (2) 305-day milk production (305D - MP), fat yield (305D-fat) and protein yield (305D-protein); (3) Somatic cell count (SCC): the annual geometric average of SCC was used in this statistical analysis, with a cutoff of ≥250,000 cells/mL indicating subclinical mastitis (25); (4) fat-to-protein ratio (FPR) in milk on the first test post-calving, with a cutoff of ≥1.5 used to indicate subclinical ketosis (26); and (5) variables reflecting reproduction performance: number of days in milk at first service (DIM-FS) and calving-to-conception interval (days open) (CCI). Cows that remained open for the lactation were removed from the analysis.

Only cows with complete data available for a period of at least one year post-testing were included in the data analysis.

### Statistical analysis

#### Hierarchical structure diagram and causal diagram

**Figure 1:**
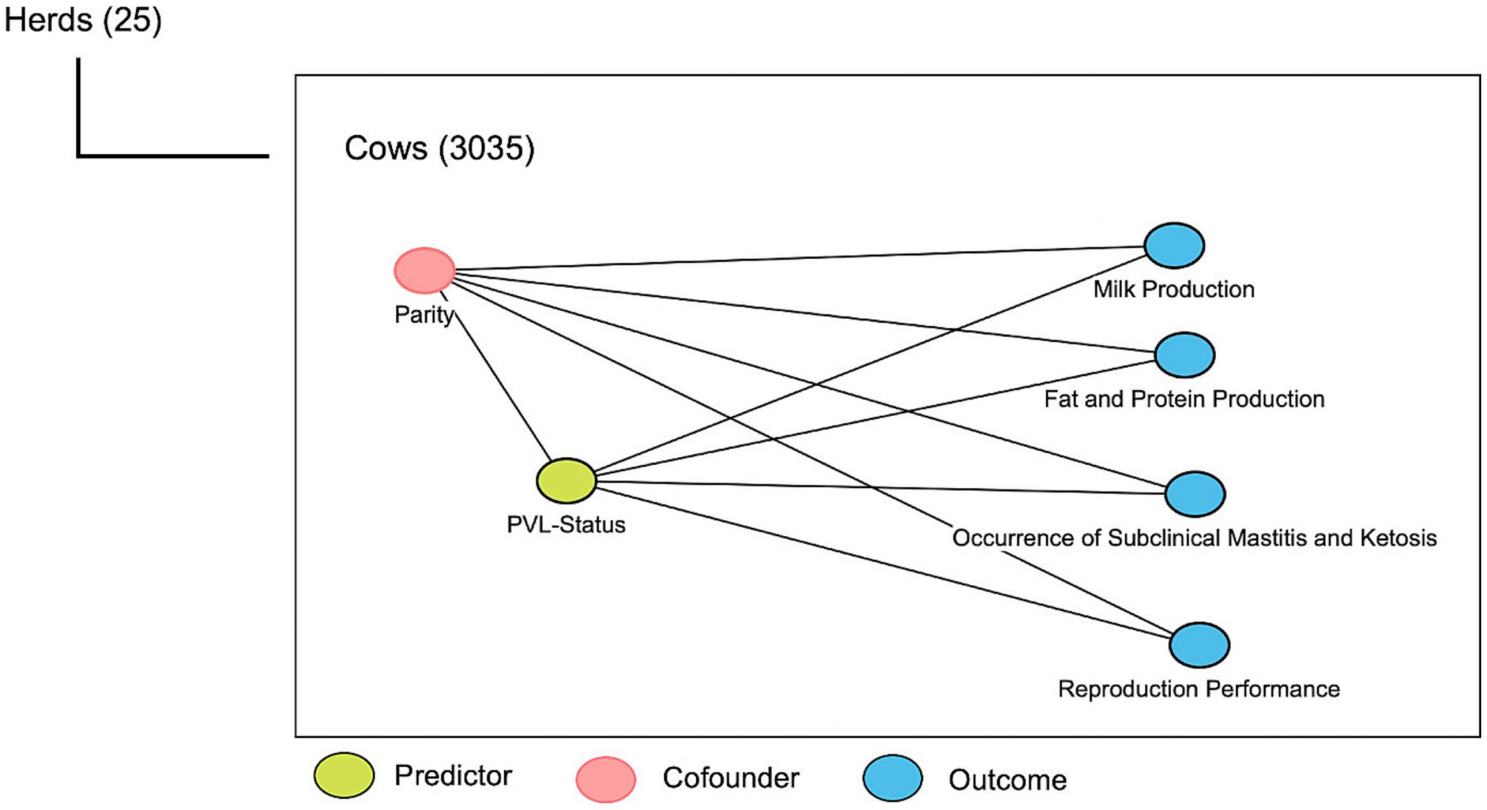
Causal diagram for the effect of proviral load (PVL) on production and reproduction parameters, as well as occurrence of subclinical diseases in 25 dairy herds. Two-level hierarchy affects the outcomes of interest.

A simplified 2-level hierarchical causal diagram was constructed to illustrate the hypothesized relationships between the explanatory and outcome variables. The explanatory variable of interest was PVL status. We had one potential confounder which was parity (Figure 1).

#### Association between PVL and cow production, reproduction performance or occurrence of subclinical diseases

Given the hierarchical structure of the collected data with cow nested in herd (Figure 1), a two-level linear mixed-effect regression model was used for continuous response variables to estimate the association between cow production indices (305D - MP, 305D-fat, 305D-protein, DIM-FS, and CCI) as outcomes of interest (continuous variables) and PVL as a main predictor.

PVL was categorized as a binary variable, as either high (≥1 copy /WBC) or low (<1 copy/WBC). This PVL cutoff value was being used in a voluntary BLV control program in the Canadian Maritime provinces and has also been shown to be the best PVL cutoff to correlate with lymphocytosis in our population (23). The population of interest was high PVL and the reference population was low PVL. Similarly, a two-level mixed effect logistic regression was used for categorical response variables to assess whether the occurrence of subclinical diseases, namely subclinical mastitis and subclinical ketosis, were associated with PVL status. Explanatory variables (PVL status, parity, subclinical mastitis and subclinical ketosis for the milk production model; PVL status, parity and subclinical ketosis for the subclinical mastitis model; and PVL status, parity and subclinical mastitis for the ketosis model) were initially assessed for unconditional association with each outcome of interest using linear regression or logistic regression for continuous and categorical variables, respectively. Only the variables with *p* ≤ 0.2 were selected for a mixed effect multivariable model regression analysis. Model building that utilized the full model and removed the least associated variable (backward elimination) was used to retain significant variables (*p* ≤ 0.05). Two-way interaction terms were assessed between PVL status and parity, as well as between PVL status and occurrence of subclinical mastitis and between PVL status and occurrence of subclinical ketosis. Interaction terms were not retained in the final model if not significant (Wald’s test >0.05) and if they did not change the coefficient of the main predictor by more than 20% when included in the model. Confounding variables were only retained in the model if removing them from the model changed the coefficient of the main exposure variable by more than 20%. All models included parity, as it was a consistent confounder and province as fixed effects and herd as a random effect. For each predictor, a Wald test was used to determine the overall P- value to assess its significance.

All above modeling analyses were repeated with BLV^−^ versus BLV^+^ with low PVL status, and again with BLV^−^ versus BLV+ with high PVL status.

All linear mixed model assumptions of normality, linearity and equal variance were assessed graphically. Model diagnostics were assessed in each model, which permitted to rule out the presence of any outliers or influential observations.

For goodness of fit of the mixed models, the R-squared and the intraclass correlation coefficients (ICC) were calculated and assessed. Finally, a contextual effect of the main predictor recorded at the cow level (PVL status) was assessed by including the herd proportions of high PVL cows in the model to identify if there was any contextual effect [whether or not the estimated effect is related to the group (herd), or context, to which the cow belongs to Dohoo et al. (27)]. The absence of any significant effect of this added herd PVL variable can be interpreted as indicating an effect of PVL that is purely at the cow level (28).

All statistical analyses were carried out in Stata 17 (StataCorp, College Station, TX. USA).

## Results

**Figure 2:**
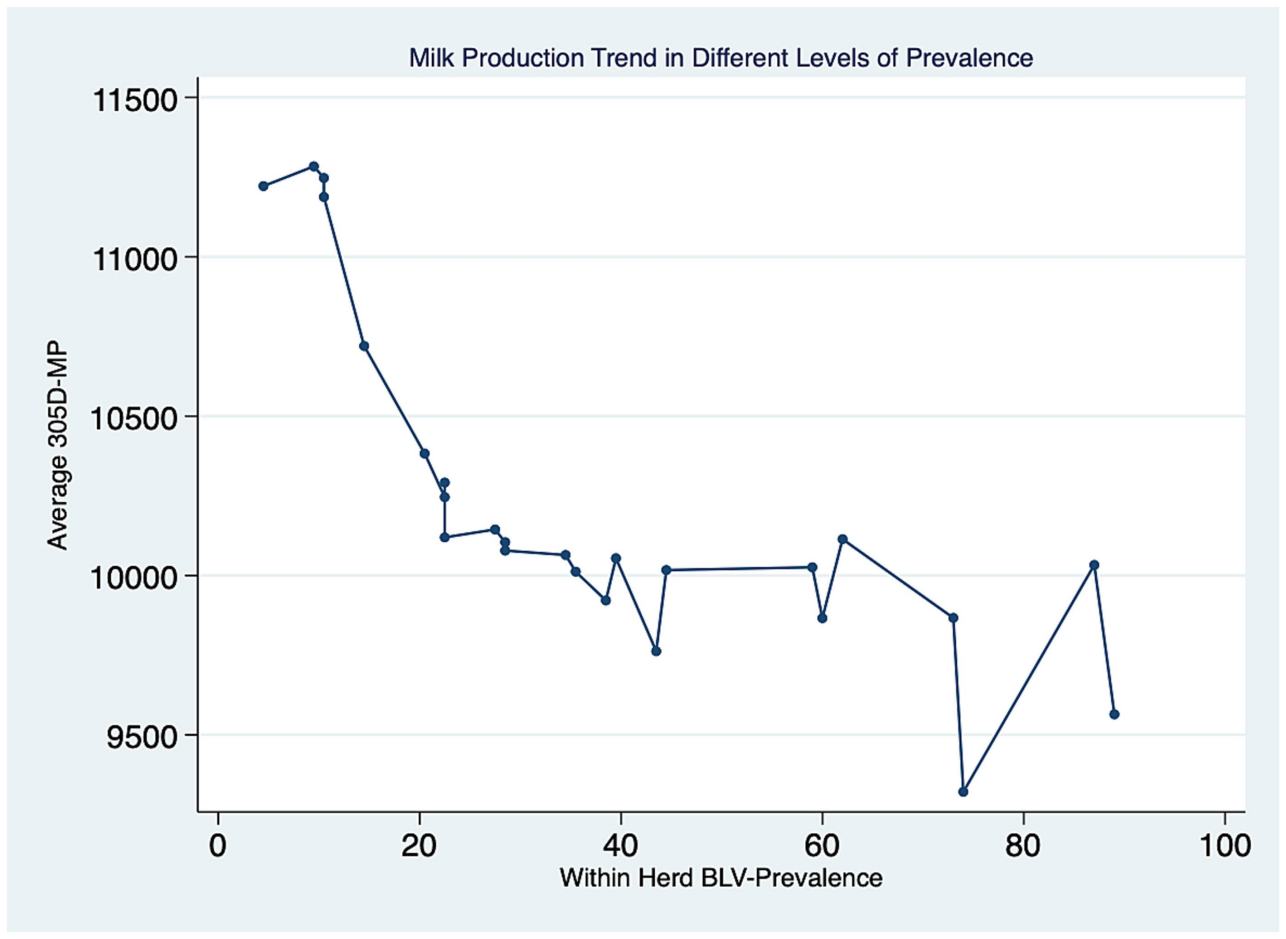
Average 305d milk production (305-MP – in kg) at the herd level for different levels of within-herd BLV prevalence (%).

**Figure 3:**
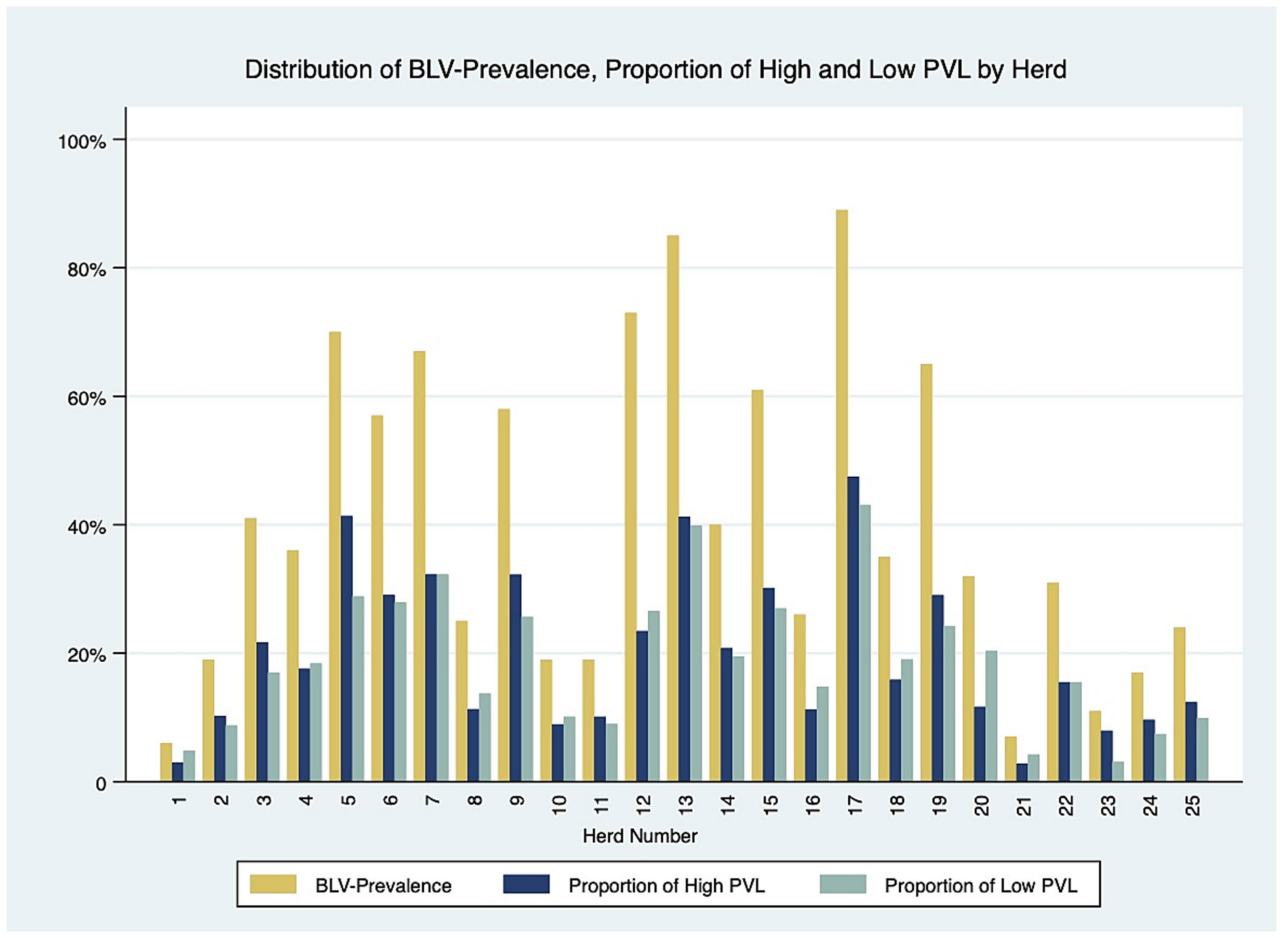
Distribution of BLV prevalence and proportion of high and low proviral load cows by herd.

Twenty-five dairy herds, four from Prince Edward Island (PEI), twelve from Nova Scotia (NS) and nine from New Brunswick (NB), were enrolled in this study, with a median herd size of 106 lactating cows [interquartile range (IQR), 71–168]. In total, 3,035 dairy cows were tested for BLV, with 1868 BLV^−^ and 1,167 BLV^+^. The overall prevalence was 38.4%. The within-herd prevalence ranged from 6 to 89%. The PVL levels measured with qPCR revealed that 624 (53%) positive cows were classified as high PVL and 543 (47%) were classified as low PVL. The median parity was 3 (IQR, 2–4). The distribution of positive cows in the parity categories was 22, 26, 21, and 31% for first, second, third and fourth or more, respectively. The mean cow’s 305d milk production was 10,221 kg (95% CI: 9878–10,565). The herd average 305-d milk production ranged from 9,321 to 11,283 kg per cow, and a trend was observed where herds with higher BLV prevalence had a lower herd average 305-d milk production per cow than herds with lower prevalence, as shown in Figure 2. There were 276 BLV^+^ cows and 252 BLV^−^ cows with subclinical mastitis; and 290 BLV^+^ cows and 507 BLV^−^ cows with subclinical ketosis. The proportion of high PVL status amongst BLV^+^ cows at the herd level varied from 2 to 47% (Figure 3). Descriptive statistics are presented in Table 1.

**Table 1:** Descriptive statistics of variables used to assess the impact of PVL on production parameters and subclinical diseases.

Variable	Mean	SD	Median	Minimum	25th percentile	75th Percentile	Maximum
PVL	1.17	1.09	1.13	0	0.11	1.78	7.1
Parity	3	1.80	3	1	2	4	11
Age (Month)	57.35	23.7	54	24	38	70	174
305 D-MP^1^	10,622	856	10,480	8,850	10,080	11,090	16,440
305D-fat^2^	434	99	424	203	361	501	689
305D-protein^3^	345	69	338	689	291	391	599
FPR^4^	1.37	0.34	1.31	0.22	1.17	1.50	2.91
CCI^5^	127	49	102	88	91	142	300
DIM-FS^6^	82	19	76	60	70	90	160
Average SCC^7^	182	324	53	44	40	189	4,404

^1^305-day milk production (kg).

^2^305-day fat yield (kg).

^3^305-day protein yield (kg).

^4Fat-to-protein ratio.^

^5Calving-to-conception interval.^

^6Day in milk at first service.^

^7^Geometric annual average somatic cell count (cells/uL).

### Relationship between PVL and milk production

PVL-status, parity and province (*p* < 0.20) were included into all models. Subclinical mastitis and subclinical ketosis were forced into the mixed multivariable linear regression model for further analysis. Inclusion of the interaction terms of PVL status with parity, occurrence of subclinical mastitis and occurrence of subclinical ketosis did not show any significance (*p* > 0.05) and did not result in different coefficients when compared to the model without interaction terms. Only parity showed a confounding effect and therefore was retained in the final models. Subclinical mastitis and subclinical ketosis did not show a confounding effect; however, they were forced in the final models because these variables often correlated with the outcome variables.

After adjusting for parity, subclinical mastitis, subclinical ketosis and herd random effects, there was a significant negative association (*p* < 0.001) between PVL status and 305D-Milk Production, 305D-fat yield, and 305D-protein yield. BLV^+^ cows with high PVL had an approximately 387 kg reduction (−4%) in 305D-Milk Production compared to BLV^+^ cows with a low PVL (*p* < 0.001) (Table 2). The ICC was 0.19 (95% CI 0.11–0.25). Similarly, a BLV^+^ cow with a high PVL had a 30 kg reduction in 305D-fat yield, and 11 kg reduction in 305D-protein yield in a lactation compared to BLV^+^ cows with low PVL.

**Table 2:** Mixed linear regression model for 305-day milk production, 305-day fat yield and 305-d protein yield and low vs. high PVL status in BLV-positive cows.

	Coefficient	[95% confidence interval]		*p*-value
**305d milk production**				
PVL-status				<0.001^*^
Low PVL	Reference			
High PVL	−387.37	−474.20	−300.54	<0.001
Province PEI	Reference			0.7
Nova-scotia	58.98	−330.53	448.50	0.3
New-Brunswick	148.20	−261.53	541.93	0.7
Parity 1	Reference			<0.001*
2	563.27	439.49	687.05	<0.001
3	728.64	598.01	859.27	<0.001
≥4	875.71	752.58	998.84	<0.001
Subclinical mastitis	−56.57	−158.78	45.63	0.3
Subclinical ketosis	−119.51	−224.58	−14.43	0.05
Intercept	10221.34	9877.96	10564.72	<0.001^*^
**305-day fat yield**				
PVL-Status				<0.001*
Low PVL	Reference			
High PVL	−30.21	−44.95	−15.47	<0.001*
Province PEI	Reference			0.9
Nova-scotia	−6.91	−91.28	77.46	0.9
New-Brunswick	−15.19	−102.47	72.08	0.7
Parity 1	Reference			<0.001*
2	46.78	25.74	67.81	<0.001
3	45.54	23.34	67.74	<0.001
≥4	57.38	36.44	78.32	<0.001
Subclinical mastitis	−6.62	−25.39	12.14	0.5
Subclinical ketosis	7.64	−10.25	25.55	0.4
Intercept	368.01	316.59	419.43	<0.001*
**305-day protein yield**				
PVL-Status				<0.001*
Low PVL	Reference			
High PVL	−11.02	−17.38	−4.66	<0.001
Province PEI	Reference		0.4	
Nova-scotia	−32.43	−79.23	14.37	0.2
New-Brunswick	−19.42	−67.93	−29.09	0.4
Parity 1	Reference			<0.001*
2	31.62	53.38	84.19	<0.001
3	46.66	72.92	108.72	<0.001
≥4	55.78	89.71	125.56	<0.001
Subclinical mastitis	−4.52	−12.01	2.96	0.2
Subclinical ketosis	−4.57	−12.30	3.16	0.2
Intercept	264.11	230.77	297.46	<0.001*

*Highly significant.

Intraclass correlation coefficient = 0.19 (305-day milk production model), 0.25 (305-day fat yield model), 0.26 (305-day protein yield model).

*R*^2^: 0.29 (305-day milk production model), 0.24 (305-day fat yield model), 0.18 (305-day protein yield model).

### Relationship between PVL and reproduction indices

Two reproduction parameters, calving-to-conception interval (CCI) and days in milk to first service (DIM-FS), were modeled independently with PVL-status (low vs. high), parity and province based on univariable analysis (*p* < 0.2). Adding the interaction term PVL-status and parity did not improve the model (*p* > 0.05) and was not retained in both models. Parity showed a confounding effect in both models. Both reproductive parameters were highly associated with PVL status. The DIM-FS was 13 days longer and the CCI was prolonged by 50 days in cows with high PVL in comparison to cows with low PVL status (*p* < 0.001) (Table 3).

**Table 3:** Mixed linear regression model for the reproduction indices, calving conception interval (CCI) and days in milk to first service (DIM-FS), and PVL status.

	Coefficient	[95% confidence interval]		*P*-value
**CCI calving-to-conception interval**				
PVL-status				<0.001*
Low PVL	Reference			
High PVL	50.42	43.88	56.96	<0.001
Province PEI	Reference			0.7
Nova-scotia	9.26	−11.64	30.17	0.4
New-Brunswick	5.66	−15.79	27.17	0.6
Parity 1	Reference			0.3
2	4.37	−5.10	13.84	0.4
3	7.33	−2.65	17.32	0.1
> = 4	8.15	−1.21	17.52	0.08
Intercept	77.61	57.98	97.24	<0.001^*^
**DIM-FS day in milk at first service**				
PVL-status	13.49			<0.001^*^
Low PVL	Reference			
High PVL	13.49	9.89	17.08	<0.001
Province PEI	Reference			0.6
Nova-scotia	0.63	−9.48	10.75	0.9
New-Brunswick	−2.76	−13.11	7.58	0.6
Parity 1	Reference			0.7
2	1.98	−3.22	7.18	0.4
3	3.12	−2.36	8.60	0.2
> = 4	1.86	−3.27	6.99	0.4
Intercept	69.99	60.28	79.70	<0.001^*^

^*^ Highly significant.

Intraclass correlation coefficient = 0.16 (Calving-to-conception model), 0.14 (day in milk at first service model).

*R*^2^ = 0.26 (Calving-to-conception model), 0.19 (day in milk at first service model).

### Relationship between PVL and occurrence of subclinical disease

A mixed effect logistic regression model was used to assess the relationship between low and high PVL in BLV^+^ cows and occurrence of two subclinical diseases. Parity and province (*p* < 0.20) were included into both models. Parity, subclinical ketosis and subclinical mastitis were assessed for confounding effects and only parity showed a confounding effect in both models. Adding the interaction term PVL-status and parity (both models), PVL-status and subclinical ketosis (for the subclinical mastitis model), and PVL-status and subclinical mastitis (for the subclinical ketosis model) did not improve both models (*p* > 0.05) and were not retained in both models. One model demonstrated that the PVL level was strongly associated with the occurrence of subclinical mastitis (*p* < 0.001) with an odds ratio estimate of 2.38 (Table 4). The occurrence of subclinical ketosis did not differ significantly between high PVL and low PVL cows (*p* = 0.3) (Table 4).

**Table 4:** Mixed effect logistic regression model for subclinical mastitis/subclinical ketosis and PVL status (low vs. high) of BLV^+^ cows.

	Odds ratio	[95% confidence interval]		*P*-value
**Subclinical mastitis**				
PVL-status				<0.001*
Low PVL	Reference			
High PVL	2.38	1.78	3.19	<0.001
Province PEI	Reference			0.05
Nova-scotia	1.22	0.72	2.06	0.4
New-Brunswick	0.78	0.46	1.34	0.4
Parity 1	Reference			0.5
2	0.93	0.62	1.40	0.7
3	1.07	0.71	1.64	0.7
> = 4	1.03	0.69	1.53	0.8
Intercept	0.18	0.10	0.33	<0.001^*^
**Ketosis**				
PVL-status				0.318
Low PVL	Reference			
High PVL	1.13	0.88	1.47	0.318
Province PEI	Reference			0.5
Nova-scotia	1.67	0.58	4.76	0.3
New-Brunswick	1.13	0.38	3.30	0.8
Parity 1	Reference			0.06
2	1.18	0.82	1.70	0.4
3	1.07	0.72	1.59	0.7
> = 4	1.58	1.09	2.29	0.01
Intercept	0.93	0.36	2.39	0.8

^*^ Highly significant.

Intraclass correlation coefficient = 0.13 (subclinical mastitis model), 0.17 (ketosis model).

The contextual effect of PVL on each production parameter was not significant. Therefore, the estimated effect of the PVL on cow’s performance parameters was not due to the between-herd management differences and there was no need to adjust the coefficient interpretation.

### Comparison between BLV- cows and BLV+ cows with either high or low PVL

When comparing BLV^−^ cows and BLV^+^ cows with high PVL, there was a significant reduction in milk, fat and protein yields (*p* < 0.006), as well as a significant increase in reproduction parameters DIM-FS and CCI (*p* < 0.0001) and increased occurrence of subclinical mastitis (*p* < 0.0001) in high PVL status cows compared to BLV^−^ cows. There was, however, no significant difference concerning the occurrence of subclinical ketosis. (Table 5).

**Table 5:** Summarized results of mixed-effect multivariable linear regression models and mixed effect multivariable logistic regression models for measuring associations between each PVL-status and BLV-negative cows and milk production, reproduction indices and occurrence of subclinical mastitis and ketosis.

		Coefficient (Odd ratio)	*P*-value	ICC
Comparison high PVL versus BLV^−^ cows	Milk production	−431.54	0.006*	0.14
	Calving conception interval	48.97	0.001*	0.15
	Days in milk first service	15.88	0.001*	0.13
	Subclinical mastitis	0.91 (OR = 2.48)	0.001*	0.12
	Subclinical ketosis	0.14 (OR = 1.15)	0.3	0.22
Comparison low PVL versus BLV^−^ cows	Milk production	33.65	0.8	0.15
	Calving conception interval	2.68	0.4	0.14
	Days in milk first service	−1.79	0.1	0.12
	Subclinical mastitis	0.28 (OR = 1.32)	0.06	0.11
	Subclinical ketosis	−0.11 (OR = 0.89)	0.4	0.18

*Highly significant.

When comparing BLV^−^ cows to BLV^+^ cows with low PVL, no difference was found for all the parameters. (Table 5).

## Discussion

This study evaluated the association of BLV PVL status with milk production and reproduction parameters, as well as the occurrence of common subclinical diseases, mastitis and ketosis, in the dairy industry.

Multiple studies have investigated the impact of BLV status on milk production. A negative association between seropositivity and milk production has been shown at the herd level in several studies (2, 29, 30). These results are in agreement with our study that showed a trend where increased BLV prevalence within a herd had decreased milk production at the herd level.

There have been contradictory results at the individual cow level, with some studies finding no association between BLV seropositivity and milk production (15, 16, 30, 31), while others did find that BLV^+^ cows have decreased milk production in comparison to BLV^−^ cows (14, 32, 33). This current study determined that there was a significant difference in milk production parameters (305-d milk yield, 305-d fat yield, and 305-d protein yield) between BLV^+^ cows with a high PVL versus BLV^+^ cows with a low PVL and versus BLV^−^ cows but not between BLV^+^ cows with a low PVL and BVL negative cows. This result is in agreement with a similar study performed in a smaller population with 9 herds in Alberta (22), although in our study, the estimated difference in milk yield was ~431 kg reduction vs. 294 kg in the Alberta study, when high PVL cows were compared to BLV^−^ cows. This difference could be due to the lower cutoff they used to distinguish high versus low PVL cows (0.5 vs. 1.0 in our study) or their smaller sample size. Our results were also in agreement with previous studies looking at associations between milk production and persistent lymphocytosis (34) or high BLV ELISA optical density values (32), both of which have been shown to correlate with high proviral loads (23, 35–37). The PVL status of a BLV^+^ cow may explain, in part, the discrepancy between previous studies looking at the impact of BLV seropositivity on milk production at the cow level, as they did not account for PVL status. Studies that had few high PVL cows in their BLV^+^ cohort would be less likely to show a change in milk production parameters.

This study used two common indices to assess reproduction performance, namely days in milk at first service (DIM-FS) and calving-to-conception interval (CCI). Our results showed that PVL status was associated with reproduction performance. This result is in contrast to a recent study that reported no evidence of an association between BLV status or high PVL and fertility in Kansas beef herds (38). However, different reproductive indices were used in that study since it was conducted in beef cattle and the high PVL cutoff was slightly lower in that study (≥ 0.9 proviral copies/host DNA) than in our study. Only a few studies found reduced reproductive efficiency (39–42) associated with BLV seropositivity, which were limited to a subset of cows with lymphocytosis or lymphosarcoma, and both of these conditions are associated with a high PVL.

Disruption of the immune function of polymorphonuclear neutrophils, as well as inflammation of the mammary gland, have been shown to occur in BLV seropositive cows and are associated with higher PVL levels (20, 43–45), which is an explanation for the increased susceptibility of high PVL cows to subclinical mastitis found in our study. Increasing PVL levels have been associated with the risk and severity of clinical mastitis (46). One study did not find any significant association between PVL status and occurrence of clinical mastitis, but this lack of an association could be due to selection bias where only cases with clinical mastitis severe enough to warrant treatment were included, or due to the low power to find an association with this small population (n = 97) (47).

This current study focused on subclinical mastitis, as this can also have an impact on milk production and is of relevance for dairy producers. A recent study showed a hazard ratio for subclinical mastitis in high PVL BLV^+^ cows to be 2.61 times higher than BLV^−^ cows, with no significant difference between BLV^+^ cows with a low PVL and BLV^−^ cows (48), which is consistent with the findings in this study. Factors at the herd level, such as hygiene measures, types of milking procedures, stall type and size, and lactation stage, can also affect the prevalence of subclinical mastitis (49–51) and were accounted for in our herd clustering effect model. In addition, we used the annual geometric average of the SCC to account for the seasonal effect.

This study is the first one to assess the effect of BLV and PVL status on occurrence of subclinical ketosis in the first month after calving. We did not find any significant association between BLV PVL status and subclinical ketosis. However, we used the inline milk fat-to- protein ratio with a cutoff value of 1.5 to categorize cows with subclinical ketosis. Although this method is a practical way for screening for subclinical ketosis at the herd level, it has limited accuracy to diagnose subclinical ketosis in individual cows, with a high false discovery rate (26). Further studies with measurement of betahydroxybutyrate concentrations in the blood or milk are warranted (52).

Multiple cutoff values with different units have been used to define high PVL cows, which makes it difficult to compare studies. Examples of cutoff values include 100,000 copies/μg of DNA (35), 500 proviral copies/50 ng of genomic DNA (53), 100,000 copies/10^5^ cells (12), and 0.5 copies/beta-actin copies (54).

There is no consensus on the most appropriate cutoff value to define high PVL BLV^+^ cows. In our study, we used a cutoff of 1.0 viral genome amplified per WBC, which is the same as 100,000 copies/10^5^cells and 0.5 copies/beta-actin copies, both used in previous publications. A previous study from our institution elaborated a statistical model to predict high proviral load using lymphocyte counts since lymphocytosis correlates with increasing PVL. In that study, it was determined that the best reliability of the model was obtained with the cutoff of 1 copy of provirus per white blood cell (23). We also used the cutoff of 1 copy per WBC for PVL in a voluntary BLV control program in the Canadian Maritime provinces. For these reasons, a cutoff value of 1 copy per WBC or greater was chosen for high PVL in this study. This cutoff seems much higher than the cutoff used in a recent study conducted in Alberta, Canada (1.0 vs. 0.25 per WBC) yet similar results were found (22). However, in that study, they calculated the PVL by dividing the number of BLV copies by the number of beta-actin copies but interpreted this ratio as copies per white blood cell which is incorrect, as the number of beta-actin copies must be first divided by 2, to account for the fact that each cell contains 2 copies of the gene (23, 55). Therefore, their cutoff of 0.25 is not per white blood cell and corresponds to a cutoff of 0.5 of copies per WBC. Similarly, another study used a cutoff of 0.5 copies per beta-actin copies, which corresponds to the cutoff used in this study of 1.0 copies per WBC (54). This equivalency illustrates the importance of having standardization in defining high PVL to be able to better compare studies’ results in the future.

Our study had some limitations inherent to milk production data collection. Selection bias might have happened, with cows with very low production possibly removed early in lactation by producers and therefore not included in the analyzed data. Survivor bias was also possible; however, we limited this bias by using data collected before the implementation of target culling of high PVL cows as part of the BLV management program. PVL is dynamic; therefore, there could have been misclassification of some cows’ status in one direction, from BLV- to BLV+ and from low PVL to high PVL. As such, the associations identified in our study could be underestimated and potentially biased toward the null. Although survival analysis can be useful for CCI (time to event outcome) in some contexts, other methods such as linear regression have been used successfully to assess CCI in dairy cattle (56). In our analysis we used CCI as a continuous variable in a mixed multiple linear regression model to assess the effect of PVL on this interval because the assumption of normal distribution of error was met and because of easier interpretation of the coefficient than survival analysis. In addition, this was a cross-sectional study with one point in time assessment of PVL and therefore, using a survival analysis that is focused on time to conception would likely be inappropriate since we do not really know when the starting point for their high PVL status began. We excluded only a few open cows because occasionally a farmer chose not to breed a cow or a cow was bred unsuccessfully during the study period. Excluding these cows in the analysis may have led to underestimating the effect and therefore bias toward the null. Finally, the test used to determine subclinical ketosis might have led to a high false discovery rate, which could have impacted our results. Further studies with measurement of beta-hydroxybutyrate concentrations in the blood would be warranted to confirm our results.

In conclusion, high BLV proviral load was associated with decreased milk production and fat and protein yields, compared to BLV^+^ cattle with a low PVL. In addition, BLV high PVL status decreased reproductive efficiency, as well as increased the risk of subclinical mastitis. This reduction in performance parameters, in addition to the higher risk of transmission of BLV from high PLV cows to naïve cows, supports the importance of identifying and culling high PVL cows in herds. This targeted culling is especially important in herds with a high BLV prevalence, where culling of all seropositive cows is not feasible.

## Data Availability

The raw data supporting the conclusions of this article will be made available by the authors, without undue reservation.
